# Quality, Microstructure, and Properties of Metal Alloys

**DOI:** 10.3390/ma16083019

**Published:** 2023-04-11

**Authors:** Tomasz Lipiński

**Affiliations:** Faculty of Technical Sciences, University of Warmia and Mazury in Olsztyn, 10-719 Olsztyn, Poland; tomaszlipinski.tl@gmail.com

In the course of evolution, humankind has used many construction materials. The first construction materials were natural materials: wood, stone, leather, etc. In the course of the development of knowledge and technology, metals were gradually used. However, their functional properties were quite low, and their use was limited. Thanks to many years of research, a number of metal alloys have been produced. Some of them, in an improved form, are produced to this day. Pure metals are not as widely used as their alloys.

Materials are produced to meet the needs of customers. Customers decide the success or failure of each of the products offered to them. When selecting the material, a number of its features and properties are taken into account. Not only are physical, chemical, and electrical properties; accessibility; susceptibility to processing expected for a specific manufacturing process; and costs taken into account, but also economic, ecological, social, and other parameters are analyzed.

The choice of material also depends to a large extent on the scale of production. In the case of unit and small-lot production, for economic reasons, it is not profitable to develop a special material and a complex manufacturing technology dedicated to the product. Thus, available materials and available manufacturing technologies are used. In mass production, it may be justified to select the material, and even its design, taking into account the specific characteristics of the product. Similarly, with production technology for large-series and mass production, it is justified to develop a special production line. When choosing the material and production technology, costs and profits, the human factor, technological and production capabilities, availability and timeliness of deliveries, material competitiveness, recyclability, and many other factors were also taken into account.

Another factor determining the success of the material is its quality. With widely available advanced technology, it is possible to produce high- and very high-quality materials. However, the concept of quality in relation to materials and the products made of them is a relative concept. In practice, it is assumed that a material of appropriate quality is one that meets the expectations of recipients. However, recipients have different needs and expectations depending on the intended use of the material. Different expectations are placed on materials intended for the production of cheap products with low properties, the failure of which does not cause large losses or safety risks. However, it is diametrically different for materials intended for the production of elements whose premature wear may cause a threat to safety or large material losses. A separate group are the so-called decorative materials. This group of materials is governed by its own laws, which are not always logically justified.

Currently, a number of methods for the production of elements from metal alloys are known. Popular methods include foundry technologies, plastic processing, machining, powder metallurgy, and many others. These technologies are supplemented, among others, by heat treatment, surface engineering, etc. The popular metal alloys include both ferrous metal alloys and non-ferrous metal alloys. The use of each of these groups depends, among other things, on the expected characteristics of the product. Despite the dynamic development of plastic-based material observed in recent years, it can be seen that constructors still willingly use metal alloys. Despite the high and constantly increasing parameters of some plastics and composites, metal alloys are still willingly used and there are no reasons to eliminate them by other engineering plastics. The main reason for their dominance over other materials when used for responsible products is that there are many years of experience in their use and thus acquired knowledge regarding their behavior in various conditions and habit, which translates into trust in this group of materials.

Modern constructions, which include, airplanes, cars, and floating structures, are built using a very wide range of construction materials; these include metal alloys, plastics, natural materials, and sintered materials. The conducted research allows for the improvement of existing materials, the development of new materials, and the expansion of knowledge about the properties of materials previously implemented for production. The knowledge and experience gained in the course of research and operation of metal alloys allow us to gradually implement modern metal alloys and methods of their production. Modern metal alloys developed in recent years, compared to traditional alloys used for many years, have higher functional properties, higher durability, higher reliability, lower production cost, lower operating cost, less complicated production technology, easier recycling, and many other properties. Of course, materials developed in recent years do not have all of these features at the same time.

It is known that the quality and strength properties of metal-based construction materials result from their microstructure, which, in turn, depends, on the chemical composition of the metal alloy and its manufacturing process, among others.

The above (very abbreviated) considerations show the need to conduct research on the quality of the microstructure and properties of metal alloys, the purpose of which is to improve the properties and reduce the production costs of already known materials, and the need to conduct research aimed at developing new solutions and new materials.

This Special Issue contains nineteen articles that present the results of research on the microstructure, quality, and properties of metal alloys. The readers were presented with the works of experienced researchers on the relationship between the microstructure and the quality and properties of metal alloys.

At research properties high strength steel [1] based on the requirements of the weldability, the influencing factors of weld heat affected zone (HAZ), and the development and application status of oxide metallurgy technology are summarized in this review. Moreover, the advantages and difficulties in the application of rare earth oxide metallurgy technology are analyzed, combined with the performance mechanism of rare earth and its formation characteristics of fine and high melting point RE inclusions with distribution dispersed in liquid steel.

Previous research [2] analyzed the mechanical properties of cold-sprayed Cr_3_C_2_-25(Ni20Cr) blended with Ni-graphite as a solid lubricant deposited on 7075 aluminum alloy substrates. The cold-sprayed coatings were evaluated for their chromium carbide and graphite content, hardness, and coefficient of friction. Analysis of the microstructure of the deposited coatings revealed that graphite as a soft and brittle component fills all voids in the coating and its quantity depends on its content in the feedstock. The experimental results show that the composition of the process gas has the greatest impact on the Cr_3_C_2_ content in the coating and the proportion of graphite in the sprayed blend directly affects its hardness. In the case of the coefficient of friction, the most significant parameters were the graphite content in the sprayed blend, the spraying distance, and process gas composition.

Microstructure and texture evolution tests during superplastic deformation of SP700 titanium alloy were carried out on SP700 (Ti-4.5Al-3V-2Mo-2Fe) titanium alloy sheets at 760 °C [3]. The microstructure characteristics were investigated to understand the deformation mechanism. The results indicated that the examined alloy showed an extremely fine grain size of ~1.3 μm and an excellent superplasticity with fracture elongation of up to 3000%. The grain size and the volume fraction of the β phase increased as the strain increased, accompanied by the elements’ diffusion. The β-stabilizing elements (Mo, Fe, and V) were mainly dissolved within the β phase and diffused from α to β phase furthermore during deformation. The increase in strain leads to the accumulation of dislocations, which results in the increase in the proportion of low angle grain boundaries by 15%. As the deformation process, the crystal of α grains rotated, and the texture changed, accompanied by the accumulation of dislocations. The phase boundary (α/β) sliding accommodated by dislocation slip was the predominant mechanism for SP700 alloy during superplastic deformation.

Using X-ray diffraction and diffraction line broadening analysis, researchers [4] quantified the effects of isothermal temperatures on the average dislocation density was assessed for different thermal dynamic driving forces in terms of activation energy and cooling rate. They stated that, as the isothermal holding temperature is increased, the dislocation density in the bainite matrix decreases from 1.55 × 1017 to 8.33 × 1015 (m^−2^) due to the reduction in the plastic deformation in the austenite in the transformation. At the same time, the activation energy required decreases only after passing the martensite and lower bainite mixed phase. A new method for better estimating the average dislocation density in bainitic steel is also proposed.

In another study [5], the effect of the Mo contents of 1.0 wt.%, 1.5 wt.%, 2.0 wt.%, and 3.0 wt.% on the microstructures and mechanical properties of the polycrystalline superalloy with a high W content was studied. The typical dendrite morphology was observed in the high-W superalloy with different Mo contents, containing γ matrix, γ′ phase, eutectic, and MC carbide. After the heat treatment, the primary MC carbides were decomposed into M6C carbides, while a needle-like topologically close-packed (TCP) phase was formed in the alloy with high Mo content, in contrast to the other three alloys with low Mo content. Researchers found improved stress fracture life at 975 °C/225 MPa due to the combination of the carbide’s suppressed micro-crack propagation and a more negative fit.

In [6], the effect of different Cu content on the reversed austenite formation, tensile strength, and impact toughness of a low-carbon martensitic stainless steel (0Cr13Ni4Mo) was systematically investigated through use of a transmission electron microscope (TEM), transmission Kikuchi diffraction (TKD), atom probe tomography (APT), and other characterization methods and mechanical property tests. The results showed that the addition of Cu decreased the phase transition temperatures of martensite and austenite and increased the volume fraction of the reversed austenite. APT results indicated that Cu-rich clusters first formed with alloying elements such as ferrum (Fe) and nickel, then grew to be precipitates through rejection of the alloying elements. The Ni atoms diffused towards the interface between the precipitates and the martensite matrix, which provided heterogeneous nucleation sites for the reversed austenite. Cu precipitations strengthened tensile strength during tempering; however, it generated temper brittleness in the steel at a tempering temperature of 450 °C.

Another study [7] examined the possibility of replacement in SmCo5 alloy of Sm with other, more abundant rare earth atoms, such as Ce-La. These raw materials are usually called “free” rare-earth minerals, appearing as a by-product during mining and processing of other raw materials. Samples with nominal stoichiometry Sm_1−x_MMxCo5 (x = 0.1–1.0) were prepared in bulk form with conventional metallurgy techniques and their basic structural and magnetic properties were examined. The materials retain the hexagonal CaCu5-type structure while minor fluctuations in unit cell parameters as observed with X-ray diffraction. Incorporation of Ce-La degrades intrinsic magnetic properties, Curie temperature drops from 920 K to 800 K across the series, and mass magnetization from 98 Am^2^/kg to 60 Am^2^/kg.

To determine the influence of the hard-facing technology on characteristics of the gears’ working surfaces, the experimental investigations were performed on samples hard-faced on the steel for cementation, by varying the filler metals (FM) and the hard-facing regimes [8]. The samples tested were hard-faced by five “hard” and three “soft” filler metals. Experimental investigations included measuring the hard-faced layers’ hardness and determination of their microstructure, and the wear resistance in the laboratory conditions, on tribometer, and on a specially designed device for tests in the real operating conditions of gears. The results obtained were compared to results of the base metal (BM) tests taking into account which filler metal and which welding procedure are optimal for regeneration of the worn teeth surfaces.

Corrosion-resistant steels were also tested. Influence of heat treatment and compared with the state without heat treatment of a part produced by the SLM (selective laser melting) method of stainless steel, 316L on microstructure, and mechanical properties were tested in [9]. Subsequently, TIG (tungsten inert gas) welds were created on the base materials processed in this way. Microstructural analysis revealed significant differences between samples with and without heat treatment. The results of these tests are supported by SEM analysis. EDAX (energy dispersive analysis of X-rays) semiquantitative analysis confirmed the presence of ultra-fine pores in the structure. The results of mechanical tests show that the solution annealing at 1040 °C for 0.5 h gives better results than the same heat treatment with a duration of 2 h.

Corrosion resistance behavior of PEP treated AISI 316L stainless steel of plasma electrolytic polished surfaces without/with chemical pretreatment (acid cleaning) was evaluated and compared with original non-treated (as received) surfaces by three independent test methods in 0.9 wt.% NaCl solution at a temperature of 37 ± 0.5 °C: electrochemical impedance spectroscopy (EIS), potentiodynamic polarization (PP), and exposure immersion test [10]. The results obtained indicated high corrosion resistance of PEP treated surfaces also without chemical pretreatment, which increases the ecological benefits of PEP technology. The authors also studied PIII-treated surfaces. The results of three independent corrosion tests consistently confirmed a significant increase in corrosion resistance after two doses of PIII nitriding [11]. Study [12] deals with the corrosion behavior of sensitized AISI 304 stainless steels in acid 1 M chloride solution (pH = 1.1) at temperatures of 20 ± 3 °C and 50 °C. The specimens after sensitization were tested as covered by high-temperature surface oxides, and after their chemical removal. The results obtained showed that sensitization significantly conditions corrosion regardless of the removal of high-temperature oxides, and the elevated temperature mainly acts as its accelerating factor.

The analysis was also carried out on foundry metal alloys. The crystallization of alloyed ductile iron (without the addition of magnesium) with oxide bifilm inclusions is discussed [13]. Based on the obtained results, a model of spheroidal graphite crystallization near bifilm inclusions was proposed. The surface of the analyzed graphite particles was smooth, which suggests a primary crystallization process. The phenomenon of simple graphite and bifilm segregation towards the heat center of the castings was also documented.

Three papers were devoted to aluminum alloys. The first research to create a unified rank model for detection methods in the identification of aluminium casting non-conformities, in line with the paradigms of the fourth industrial revolution was presented at [14]. The resulting ranking of detection methods indicated the NDT method as the most effective, which was influenced by the significant detection of critical non-conformities and the automation of the process.

In study [15], an efficient design of a Ti-modified Al-Si-Mg-Sr casting alloy with simultaneously enhanced strength and ductility was achieved. High-throughput Scheil-Gulliver solidification simulation of the A356-0.005Sr alloy with different Ti contents was carried out to establish the “composition-microstructure” quantitative relationship of the alloy. Experimental data within the Bayesian optimization framework, the relationship “composition/processing-microstructure-properties” of A356-0.005Sr with different Ti contents was constructed and validated. The present integration method may serve as a general one for the efficient design of casting alloys, especially in the high-dimensional composition space.

Study [16] presents the investigation of the effect of heat treatment on mechanical properties (tensile strength Rm, elongation A5, and hardness HBW) of the AlSi7Mg alloy without modification and modified with strontium. Obtained results allowed us to determine T6 heat treatment parameters associated with the improvement of mechanical properties of the alloy with simultaneous limitation of duration of solutioning and aging treatments. Modification of the alloy using strontium before heat treatment facilitates the process of fragmentation and balling of silicone precipitates.

A new low-melting-point brazing filler metal Al-5.0Si-20.5Cu-2.0Ni was prepared by using melt-spinning technology, then applied to CAB of 3003 aluminum alloy in [17]. The microstructure of brazing filler metal was uniform, and the grain size was less than 500 nm. As the brazing temperature reached 575 °C, the void in the joint disappeared completely. The morphology of CuAl_2_ was sensitive to the brazing temperature and dwell time. The appearance of net-like CuAl2 brazed at 575 °C for 20 min was more beneficial to improve joint mechanical properties. The leakage rate of the joint was qualified to be 10−10 Pa·m^3^/s when the brazing temperature was 570 °C or higher. The maximum shear strength of 76.1 MPa can be obtained when the joint was brazed at 575 °C for 20 min.

Research conducted on an industrial scale on low-carbon and medium-carbon steel was also presented [18,19]. Study [18] presents the bending fatigue strength of steel hardened and tempered at different temperatures, subject to the relative volume of inclusions. The research shows that the main fraction of non-metallic inclusions is Al_2_O_3_, the most numerous were impurities with a diameter of less than 2 µm, argon refining does not affect the proportion of non-metallic inclusions of large dimensions (with a diameter of over 15 µm), and the influence of non-metallic inclusions on the strength of the steel is also related to the microstructure of the steel constituting the matrix of inclusions. Study [19] describes the properties of steel presented by the dispersion index calculated on the basis of the average distance between the impurity and the average impurity size. This parameter makes it possible to estimate the fatigue strength of steel, taking into account the size of impurities and the distance between these impurities. The paper attempted to determine the scatter index and its impact on the fatigue resistance coefficient for steel.

The work presented in this Special Issue [1,2,3,4,5,6,7,8,9,10,11,12,13,14,15,16,17,18,19] demonstrates the possibilities of further improving the microstructure quality and mechanical properties of metal alloys.
